# 8b,8c-Diphenyl-2,6-bis(4-pyridyl­meth­yl)­perhydro-2,3a,4a,6,7a,8a-hexa­aza­cyclo­penta­[*def*]fluorene-4,8-dithione chloro­form solvate

**DOI:** 10.1107/S1600536810020040

**Published:** 2010-06-05

**Authors:** Cong Deng, Wenming Shu, Dongxue Zhang

**Affiliations:** a Key Laboratory of Pesticides and Chemical Biology of the Ministry of Education, College of Chemistry, Central China Normal University, Wuhan 430079, People’s Republic of China

## Abstract

In the thio­glycoluril system of the title compound, C_32_H_30_N_8_S_2_·CHCl_3_, the two pyridine rings are roughly parallel, forming a dihedral angle of 7.2 (1)°, and the distance between the centroids of the two phenyl rings is 3.951 (5) Å. The chloro­form solvent mol­ecule is linked to the main mol­ecule *via* a weak C—H⋯N hydrogen bond.

## Related literature

For applications of glycoluril derivatives, see: Rowan *et al.* (1999 ▶). For the preparation of the title compound, see: Broan *et al.* (1989 ▶); Li *et al.* (2008 ▶). 
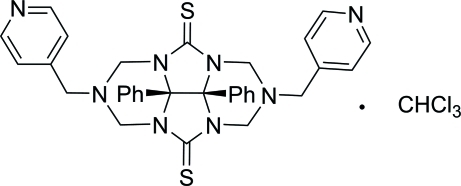


## Experimental

### 

#### Crystal data

C_32_H_30_N_8_S_2_·CHCl_3_
*M* *_r_* = 710.13
Triclinic, 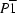

*a* = 9.5381 (6) Å
*b* = 12.1712 (8) Å
*c* = 14.8765 (9) Å
α = 100.978 (1)°
β = 91.699 (1)°
γ = 98.500 (1)°
*V* = 1673.81 (18) Å^3^
*Z* = 2
Mo *K*α radiation
μ = 0.44 mm^−1^
*T* = 294 K
0.20 × 0.10 × 0.10 mm

#### Data collection

Bruker SMART APEX CCD area-detector diffractometer
11061 measured reflections
5690 independent reflections
2136 reflections with *I* > 2σ(*I*)
*R*_int_ = 0.109

#### Refinement

*R*[*F* ^2^ > 2σ(*F* ^2^)] = 0.062
*wR*(*F* ^2^) = 0.210
*S* = 0.85
5690 reflections
415 parameters
H-atom parameters constrained
Δρ_max_ = 0.47 e Å^−3^
Δρ_min_ = −0.39 e Å^−3^

### 

Data collection: *SMART* (Bruker, 1997 ▶); cell refinement: *SAINT* (Bruker, 1999 ▶); data reduction: *SAINT*; program(s) used to solve structure: *SHELXS97* (Sheldrick, 2008 ▶); program(s) used to refine structure: *SHELXL97* (Sheldrick, 2008 ▶); molecular graphics: *SHELXTL* (Sheldrick, 2008 ▶); software used to prepare material for publication: *SHELXTL*.

## Supplementary Material

Crystal structure: contains datablocks I, global. DOI: 10.1107/S1600536810020040/cv2714sup1.cif

Structure factors: contains datablocks I. DOI: 10.1107/S1600536810020040/cv2714Isup2.hkl

Additional supplementary materials:  crystallographic information; 3D view; checkCIF report

## Figures and Tables

**Table 1:** Hydrogen-bond geometry (Å, °)

*D*—H⋯*A*	*D*—H	H⋯*A*	*D*⋯*A*	*D*—H⋯*A*
C33—H33⋯N8^i^	0.98	2.33	3.168 (9)	142 (8)

Symmetry code: (i) 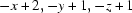.

**Fig. 1.**
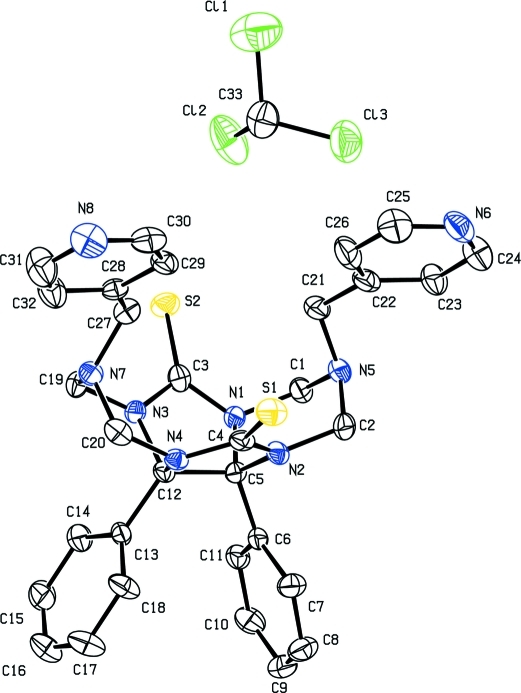
A view of (I), showing the atom-labelling scheme, with displacement ellipsoids drawn at the 30% probability level.

## supplementary crystallographic information

### Comment

Recently, molecular clips based on concave glycoluril unit have been widely investigated in supramolecular chemistry (Rowan *et al.*, 1999). We report here the structure of the title compound (Fig. 1), which is a derivative of thioglycoluril with two pyridine units. We believe the title compound would offer the possibility in construction of coordination framework with novel patterns (Li *et al.*, 2008). The crystal packing exhibits weak intermolecular C—H···N hydrogen bond (Table 1) between the chloroform solvent molecule and the main molecule.

### Experimental

The title compound was synthesized according to the literature (Broan *et al.*, 1989; Li *et al.*, 2008). Crystals of (I) suitable for X-ray diffraction were grown by slow evaporation of a chloroform-methanol (1:2) solution of the title compound under 293 K.

### Refinement

All H atoms were positioned in geometrically idealized positions and constrained to ride on their parent atoms, with C—H distances in the range 0.93-0.98 Å and U_iso_(H) = 1.2-1.5U_eq_(C). The low ratio observed/unique reflections (0.38) was mainly caused by poor quality of the crystal selected for measurements.

### Crystal data

**Table:**

C_32_H_30_N_8_S_2_·CHCl_3_	*V* = 1673.81 (18) Å^3^
*M**_r_* = 710.13	*Z* = 2
Triclinic, *P*1	*F*(000) = 736
Hall symbol: -P 1	*D*_x_ = 1.409 Mg m^−^^3^
*a* = 9.5381 (6) Å	Mo *K*α radiation, λ = 0.71073 Å
*b* = 12.1712 (8) Å	µ = 0.44 mm^−^^1^
*c* = 14.8765 (9) Å	*T* = 294 K
α = 100.978 (1)°	Block, colourless
β = 91.699 (1)°	0.20 × 0.10 × 0.10 mm
γ = 98.500 (1)°	

### Data collection

**Table:**

Bruker SMART APEX CCD area-detector diffractometer	2136 reflections with *I* > 2σ(*I*)
Radiation source: fine-focus sealed tube	*R*_int_ = 0.109
graphite	θ_max_ = 25.0°, θ_min_ = 1.7°
phi and ω scans	*h* = −11→11
11061 measured reflections	*k* = −14→13
5690 independent reflections	*l* = −17→17

### Refinement

**Table:**

Refinement on *F*^2^	Primary atom site location: structure-invariant direct methods
Least-squares matrix: full	Secondary atom site location: difference Fourier map
*R*[*F*^2^ > 2σ(*F*^2^)] = 0.062	Hydrogen site location: inferred from neighbouring sites
*wR*(*F*^2^) = 0.210	H-atom parameters constrained
*S* = 0.85	*w* = 1/[σ^2^(*F*_o_^2^) + (0.1041*P*)^2^] where *P* = (*F*_o_^2^ + 2*F*_c_^2^)/3
5690 reflections	(Δ/σ)_max_ = 0.001
415 parameters	Δρ_max_ = 0.47 e Å^−^^3^
0 restraints	Δρ_min_ = −0.39 e Å^−^^3^

### Special details

**Table:**

Geometry. All esds (except the esd in the dihedral angle between two l.s. planes) are estimated using the full covariance matrix. The cell esds are taken into account individually in the estimation of esds in distances, angles and torsion angles; correlations between esds in cell parameters are only used when they are defined by crystal symmetry. An approximate (isotropic) treatment of cell esds is used for estimating esds involving l.s. planes.
Refinement. Refinement of *F*^2^ against ALL reflections. The weighted *R*-factor wR and goodness of fit *S* are based on *F*^2^, conventional *R*-factors *R* are based on *F*, with *F* set to zero for negative *F*^2^. The threshold expression of *F*^2^ > σ(*F*^2^) is used only for calculating *R*-factors(gt) etc. and is not relevant to the choice of reflections for refinement. *R*-factors based on *F*^2^ are statistically about twice as large as those based on *F*, and *R*- factors based on ALL data will be even larger. Because of the poor quality of crystal, the ratio of Observed/Unique Reflections is 38%.

### Fractional atomic coordinates and isotropic or equivalent isotropic displacement parameters (Å^2^)

**Table:**

	*x*	*y*	*z*	*U*_iso_*/*U*_eq_	
C1	0.4638 (5)	0.4696 (4)	0.1361 (4)	0.0426 (14)	
H1A	0.4056	0.4515	0.0790	0.051*	
H1B	0.4486	0.5435	0.1685	0.051*	
C2	0.6423 (6)	0.3623 (4)	0.0735 (4)	0.0461 (15)	
H2A	0.7434	0.3659	0.0658	0.055*	
H2B	0.5934	0.3385	0.0133	0.055*	
C3	0.4314 (5)	0.4077 (4)	0.2860 (4)	0.0395 (14)	
C4	0.6795 (5)	0.2382 (4)	0.1877 (4)	0.0391 (14)	
C5	0.4489 (5)	0.2700 (4)	0.1553 (3)	0.0295 (12)	
C6	0.3463 (5)	0.2069 (4)	0.0750 (3)	0.0344 (13)	
C7	0.3908 (6)	0.1281 (5)	0.0063 (4)	0.0518 (16)	
H7	0.4868	0.1222	0.0040	0.062*	
C8	0.2952 (6)	0.0585 (5)	−0.0584 (4)	0.0565 (17)	
H8	0.3262	0.0045	−0.1031	0.068*	
C9	0.1541 (7)	0.0688 (5)	−0.0572 (4)	0.0590 (17)	
H9	0.0895	0.0223	−0.1014	0.071*	
C10	0.1082 (6)	0.1478 (5)	0.0096 (4)	0.0546 (16)	
H10	0.0125	0.1551	0.0104	0.065*	
C11	0.2042 (6)	0.2160 (4)	0.0753 (4)	0.0436 (15)	
H11	0.1725	0.2690	0.1205	0.052*	
C12	0.4498 (5)	0.2132 (4)	0.2406 (3)	0.0322 (13)	
C13	0.3367 (5)	0.1100 (4)	0.2397 (3)	0.0351 (13)	
C14	0.2062 (6)	0.1236 (5)	0.2759 (4)	0.0567 (17)	
H14	0.1877	0.1954	0.3020	0.068*	
C15	0.1037 (7)	0.0290 (6)	0.2726 (4)	0.0675 (19)	
H15	0.0167	0.0376	0.2975	0.081*	
C16	0.1289 (7)	−0.0762 (6)	0.2335 (5)	0.077 (2)	
H16	0.0588	−0.1389	0.2304	0.093*	
C17	0.2577 (7)	−0.0896 (5)	0.1986 (5)	0.072 (2)	
H17	0.2765	−0.1614	0.1730	0.086*	
C18	0.3601 (6)	0.0048 (5)	0.2017 (4)	0.0570 (17)	
H18	0.4470	−0.0045	0.1770	0.068*	
C19	0.4813 (6)	0.2929 (4)	0.4068 (3)	0.0455 (15)	
H19A	0.4783	0.3621	0.4511	0.055*	
H19B	0.4162	0.2327	0.4245	0.055*	
C20	0.6388 (6)	0.1716 (4)	0.3368 (4)	0.0469 (15)	
H20A	0.5837	0.1033	0.3497	0.056*	
H20B	0.7376	0.1611	0.3364	0.056*	
C21	0.7120 (6)	0.5325 (5)	0.1933 (4)	0.0530 (16)	
H21A	0.7130	0.4839	0.2379	0.064*	
H21B	0.6786	0.6012	0.2227	0.064*	
C22	0.8592 (6)	0.5619 (4)	0.1653 (4)	0.0482 (16)	
C23	0.8900 (6)	0.6050 (5)	0.0882 (4)	0.0577 (17)	
H23	0.8157	0.6157	0.0505	0.069*	
C24	1.0254 (7)	0.6325 (5)	0.0652 (5)	0.068 (2)	
H24	1.0400	0.6619	0.0124	0.081*	
C25	1.1118 (7)	0.5806 (6)	0.1887 (5)	0.073 (2)	
H25	1.1882	0.5730	0.2261	0.088*	
C26	0.9754 (8)	0.5499 (6)	0.2152 (5)	0.075 (2)	
H26	0.9635	0.5204	0.2682	0.090*	
C27	0.7389 (6)	0.3621 (5)	0.4180 (4)	0.0481 (15)	
H27A	0.7105	0.4275	0.4570	0.058*	
H27B	0.7554	0.3804	0.3582	0.058*	
C28	0.8721 (5)	0.3375 (4)	0.4579 (4)	0.0384 (14)	
C29	1.0046 (6)	0.3720 (4)	0.4278 (4)	0.0505 (16)	
H29	1.0126	0.4087	0.3784	0.061*	
C30	1.1231 (6)	0.3516 (5)	0.4715 (5)	0.0576 (18)	
H30	1.2098	0.3761	0.4491	0.069*	
C31	0.9976 (8)	0.2679 (6)	0.5694 (5)	0.084 (2)	
H31	0.9927	0.2311	0.6189	0.101*	
C32	0.8720 (7)	0.2838 (6)	0.5309 (5)	0.0676 (19)	
H32	0.7865	0.2582	0.5542	0.081*	
C33	0.6754 (7)	0.8554 (6)	0.3853 (5)	0.076 (2)	
H33	0.6949	0.7839	0.4001	0.091*	
Cl1	0.7802 (3)	0.9693 (2)	0.4618 (2)	0.1451 (11)	
Cl2	0.4992 (2)	0.86364 (19)	0.39764 (19)	0.1222 (10)	
Cl3	0.7234 (2)	0.86119 (18)	0.27531 (15)	0.1061 (8)	
N1	0.4182 (4)	0.3843 (3)	0.1924 (3)	0.0370 (11)	
N2	0.5961 (4)	0.2785 (3)	0.1304 (3)	0.0380 (12)	
N3	0.4351 (4)	0.3080 (3)	0.3151 (3)	0.0349 (11)	
N4	0.5925 (4)	0.1866 (3)	0.2456 (3)	0.0342 (11)	
N5	0.6126 (5)	0.4746 (4)	0.1154 (3)	0.0446 (12)	
N6	1.1383 (6)	0.6195 (4)	0.1144 (5)	0.0697 (17)	
N7	0.6238 (4)	0.2652 (3)	0.4087 (3)	0.0388 (11)	
N8	1.1278 (6)	0.3010 (5)	0.5420 (5)	0.0756 (17)	
S1	0.85378 (14)	0.24229 (12)	0.18583 (10)	0.0545 (5)	
S2	0.43168 (16)	0.53272 (12)	0.35358 (10)	0.0527 (5)	

### Atomic displacement parameters (Å^2^)

**Table:**

	*U*^11^	*U*^22^	*U*^33^	*U*^12^	*U*^13^	*U*^23^
C1	0.042 (3)	0.042 (3)	0.044 (3)	0.002 (3)	0.006 (3)	0.013 (3)
C2	0.042 (3)	0.056 (4)	0.040 (3)	0.002 (3)	0.015 (3)	0.009 (3)
C3	0.026 (3)	0.050 (4)	0.046 (4)	0.009 (3)	0.014 (3)	0.012 (3)
C4	0.038 (3)	0.028 (3)	0.046 (3)	0.004 (3)	0.014 (3)	−0.006 (2)
C5	0.025 (3)	0.032 (3)	0.031 (3)	0.006 (2)	0.004 (2)	0.003 (2)
C6	0.039 (3)	0.032 (3)	0.032 (3)	0.001 (2)	0.010 (3)	0.006 (2)
C7	0.039 (3)	0.055 (4)	0.052 (4)	−0.002 (3)	0.013 (3)	−0.009 (3)
C8	0.047 (4)	0.063 (4)	0.047 (4)	0.000 (3)	0.006 (3)	−0.012 (3)
C9	0.061 (4)	0.058 (4)	0.046 (4)	−0.009 (3)	−0.003 (3)	−0.005 (3)
C10	0.034 (3)	0.055 (4)	0.072 (5)	−0.003 (3)	−0.002 (3)	0.014 (3)
C11	0.042 (3)	0.040 (3)	0.046 (4)	0.002 (3)	0.014 (3)	0.005 (3)
C12	0.026 (3)	0.032 (3)	0.037 (3)	0.001 (2)	0.007 (2)	0.005 (2)
C13	0.035 (3)	0.038 (3)	0.034 (3)	−0.002 (3)	0.000 (3)	0.015 (2)
C14	0.050 (4)	0.060 (4)	0.054 (4)	−0.006 (3)	0.018 (3)	0.007 (3)
C15	0.046 (4)	0.090 (5)	0.058 (4)	−0.019 (4)	0.009 (3)	0.013 (4)
C16	0.066 (5)	0.066 (5)	0.091 (5)	−0.026 (4)	0.008 (4)	0.020 (4)
C17	0.066 (5)	0.038 (4)	0.104 (6)	−0.006 (3)	−0.002 (4)	0.007 (4)
C18	0.044 (4)	0.039 (4)	0.083 (5)	−0.007 (3)	0.009 (3)	0.009 (3)
C19	0.053 (4)	0.047 (3)	0.036 (3)	0.006 (3)	0.014 (3)	0.008 (3)
C20	0.038 (3)	0.039 (3)	0.066 (4)	0.008 (3)	0.008 (3)	0.013 (3)
C21	0.050 (4)	0.047 (4)	0.053 (4)	−0.010 (3)	0.012 (3)	0.002 (3)
C22	0.039 (4)	0.040 (3)	0.060 (4)	−0.006 (3)	0.006 (3)	0.004 (3)
C23	0.041 (4)	0.067 (4)	0.066 (4)	0.001 (3)	0.012 (3)	0.022 (3)
C24	0.055 (4)	0.070 (5)	0.086 (5)	0.008 (4)	0.010 (4)	0.034 (4)
C25	0.034 (4)	0.101 (6)	0.083 (5)	0.000 (4)	−0.019 (4)	0.026 (5)
C26	0.065 (5)	0.082 (5)	0.077 (5)	−0.017 (4)	0.012 (4)	0.032 (4)
C27	0.051 (4)	0.048 (4)	0.044 (4)	0.008 (3)	0.002 (3)	0.005 (3)
C28	0.030 (3)	0.040 (3)	0.040 (3)	0.003 (3)	−0.006 (3)	0.000 (3)
C29	0.040 (4)	0.047 (4)	0.057 (4)	−0.007 (3)	0.009 (3)	0.002 (3)
C30	0.025 (3)	0.064 (4)	0.075 (5)	0.006 (3)	−0.008 (3)	−0.006 (4)
C31	0.069 (5)	0.110 (6)	0.084 (6)	0.004 (5)	−0.016 (5)	0.056 (5)
C32	0.042 (4)	0.085 (5)	0.078 (5)	−0.004 (4)	0.007 (4)	0.031 (4)
C33	0.083 (5)	0.081 (5)	0.075 (5)	0.032 (4)	0.004 (4)	0.029 (4)
Cl1	0.178 (3)	0.1084 (19)	0.139 (2)	0.0305 (19)	−0.036 (2)	0.0012 (17)
Cl2	0.0895 (16)	0.1205 (19)	0.178 (2)	0.0245 (14)	0.0594 (16)	0.0679 (17)
Cl3	0.1197 (18)	0.1078 (16)	0.0969 (16)	0.0181 (14)	0.0459 (14)	0.0292 (12)
N1	0.041 (3)	0.034 (2)	0.034 (3)	0.003 (2)	0.012 (2)	0.0037 (19)
N2	0.029 (3)	0.039 (3)	0.044 (3)	−0.001 (2)	0.013 (2)	0.005 (2)
N3	0.033 (2)	0.038 (3)	0.032 (3)	0.003 (2)	0.011 (2)	0.0018 (19)
N4	0.028 (2)	0.032 (2)	0.040 (3)	0.0028 (19)	0.003 (2)	0.0026 (19)
N5	0.041 (3)	0.044 (3)	0.046 (3)	−0.004 (2)	0.011 (2)	0.010 (2)
N6	0.062 (4)	0.056 (3)	0.090 (5)	−0.003 (3)	0.027 (4)	0.017 (3)
N7	0.030 (2)	0.043 (3)	0.042 (3)	0.003 (2)	0.002 (2)	0.008 (2)
N8	0.057 (4)	0.079 (4)	0.095 (5)	0.019 (3)	−0.005 (4)	0.021 (4)
S1	0.0312 (8)	0.0584 (10)	0.0680 (11)	0.0046 (7)	0.0157 (8)	−0.0022 (8)
S2	0.0588 (10)	0.0434 (9)	0.0501 (9)	0.0087 (8)	0.0122 (8)	−0.0072 (7)

### Geometric parameters (Å, °)

**Table:**

C1—N5	1.456 (6)	C18—H18	0.9300
C1—N1	1.479 (6)	C19—N7	1.449 (6)
C1—H1A	0.9700	C19—N3	1.474 (6)
C1—H1B	0.9700	C19—H19A	0.9700
C2—N5	1.462 (6)	C19—H19B	0.9700
C2—N2	1.472 (6)	C20—N7	1.434 (6)
C2—H2A	0.9700	C20—N4	1.466 (7)
C2—H2B	0.9700	C20—H20A	0.9700
C3—N1	1.366 (6)	C20—H20B	0.9700
C3—N3	1.368 (6)	C21—N5	1.471 (7)
C3—S2	1.656 (5)	C21—C22	1.488 (7)
C4—N2	1.355 (6)	C21—H21A	0.9700
C4—N4	1.386 (6)	C21—H21B	0.9700
C4—S1	1.657 (5)	C22—C26	1.357 (8)
C5—N2	1.457 (5)	C22—C23	1.372 (8)
C5—N1	1.470 (5)	C23—C24	1.356 (8)
C5—C6	1.527 (7)	C23—H23	0.9300
C5—C12	1.557 (7)	C24—N6	1.329 (8)
C6—C11	1.376 (6)	C24—H24	0.9300
C6—C7	1.385 (6)	C25—N6	1.300 (8)
C7—C8	1.373 (7)	C25—C26	1.390 (8)
C7—H7	0.9300	C25—H25	0.9300
C8—C9	1.370 (7)	C26—H26	0.9300
C8—H8	0.9300	C27—N7	1.469 (6)
C9—C10	1.374 (7)	C27—C28	1.481 (7)
C9—H9	0.9300	C27—H27A	0.9700
C10—C11	1.377 (7)	C27—H27B	0.9700
C10—H10	0.9300	C28—C32	1.369 (8)
C11—H11	0.9300	C28—C29	1.386 (7)
C12—N4	1.448 (6)	C29—C30	1.365 (8)
C12—N3	1.467 (5)	C29—H29	0.9300
C12—C13	1.528 (6)	C30—N8	1.316 (8)
C13—C18	1.350 (7)	C30—H30	0.9300
C13—C14	1.391 (6)	C31—N8	1.348 (8)
C14—C15	1.389 (8)	C31—C32	1.368 (9)
C14—H14	0.9300	C31—H31	0.9300
C15—C16	1.361 (8)	C32—H32	0.9300
C15—H15	0.9300	C33—Cl2	1.711 (6)
C16—C17	1.367 (8)	C33—Cl3	1.724 (7)
C16—H16	0.9300	C33—Cl1	1.772 (8)
C17—C18	1.386 (7)	C33—H33	0.9800
C17—H17	0.9300		
			
N5—C1—N1	112.5 (4)	N7—C20—H20A	108.9
N5—C1—H1A	109.1	N4—C20—H20A	108.9
N1—C1—H1A	109.1	N7—C20—H20B	108.9
N5—C1—H1B	109.1	N4—C20—H20B	108.9
N1—C1—H1B	109.1	H20A—C20—H20B	107.7
H1A—C1—H1B	107.8	N5—C21—C22	112.7 (4)
N5—C2—N2	111.2 (4)	N5—C21—H21A	109.0
N5—C2—H2A	109.4	C22—C21—H21A	109.0
N2—C2—H2A	109.4	N5—C21—H21B	109.0
N5—C2—H2B	109.4	C22—C21—H21B	109.0
N2—C2—H2B	109.4	H21A—C21—H21B	107.8
H2A—C2—H2B	108.0	C26—C22—C23	114.0 (6)
N1—C3—N3	108.0 (4)	C26—C22—C21	122.6 (6)
N1—C3—S2	126.4 (4)	C23—C22—C21	123.4 (6)
N3—C3—S2	125.4 (4)	C24—C23—C22	122.0 (6)
N2—C4—N4	108.0 (4)	C24—C23—H23	119.0
N2—C4—S1	126.5 (4)	C22—C23—H23	119.0
N4—C4—S1	125.4 (5)	N6—C24—C23	123.4 (6)
N2—C5—N1	108.9 (4)	N6—C24—H24	118.3
N2—C5—C6	111.8 (3)	C23—C24—H24	118.3
N1—C5—C6	112.8 (4)	N6—C25—C26	123.5 (7)
N2—C5—C12	103.1 (4)	N6—C25—H25	118.2
N1—C5—C12	103.9 (3)	C26—C25—H25	118.2
C6—C5—C12	115.6 (4)	C22—C26—C25	121.4 (6)
C11—C6—C7	118.3 (5)	C22—C26—H26	119.3
C11—C6—C5	120.8 (4)	C25—C26—H26	119.3
C7—C6—C5	120.4 (4)	N7—C27—C28	111.6 (4)
C8—C7—C6	120.9 (5)	N7—C27—H27A	109.3
C8—C7—H7	119.5	C28—C27—H27A	109.3
C6—C7—H7	119.5	N7—C27—H27B	109.3
C9—C8—C7	120.0 (5)	C28—C27—H27B	109.3
C9—C8—H8	120.0	H27A—C27—H27B	108.0
C7—C8—H8	120.0	C32—C28—C29	115.7 (6)
C8—C9—C10	119.9 (6)	C32—C28—C27	121.4 (5)
C8—C9—H9	120.0	C29—C28—C27	122.8 (5)
C10—C9—H9	120.0	C30—C29—C28	119.3 (6)
C9—C10—C11	119.8 (5)	C30—C29—H29	120.3
C9—C10—H10	120.1	C28—C29—H29	120.3
C11—C10—H10	120.1	N8—C30—C29	126.9 (6)
C6—C11—C10	121.0 (5)	N8—C30—H30	116.5
C6—C11—H11	119.5	C29—C30—H30	116.5
C10—C11—H11	119.5	N8—C31—C32	125.7 (7)
N4—C12—N3	109.6 (4)	N8—C31—H31	117.2
N4—C12—C13	112.3 (4)	C32—C31—H31	117.2
N3—C12—C13	112.3 (3)	C31—C32—C28	120.0 (6)
N4—C12—C5	103.0 (3)	C31—C32—H32	120.0
N3—C12—C5	101.7 (4)	C28—C32—H32	120.0
C13—C12—C5	116.9 (4)	Cl2—C33—Cl3	112.1 (4)
C18—C13—C14	119.0 (5)	Cl2—C33—Cl1	109.7 (4)
C18—C13—C12	120.8 (4)	Cl3—C33—Cl1	108.3 (4)
C14—C13—C12	120.2 (4)	Cl2—C33—H33	108.9
C15—C14—C13	119.3 (5)	Cl3—C33—H33	108.9
C15—C14—H14	120.4	Cl1—C33—H33	108.9
C13—C14—H14	120.4	C3—N1—C5	111.1 (4)
C16—C15—C14	120.8 (6)	C3—N1—C1	123.0 (4)
C16—C15—H15	119.6	C5—N1—C1	114.7 (3)
C14—C15—H15	119.6	C4—N2—C5	112.7 (4)
C15—C16—C17	119.8 (6)	C4—N2—C2	126.8 (4)
C15—C16—H16	120.1	C5—N2—C2	116.3 (4)
C17—C16—H16	120.1	C3—N3—C12	113.4 (4)
C16—C17—C18	119.5 (6)	C3—N3—C19	127.7 (4)
C16—C17—H17	120.3	C12—N3—C19	114.3 (4)
C18—C17—H17	120.3	C4—N4—C12	111.8 (4)
C13—C18—C17	121.6 (5)	C4—N4—C20	124.5 (4)
C13—C18—H18	119.2	C12—N4—C20	113.6 (4)
C17—C18—H18	119.2	C1—N5—C2	110.7 (4)
N7—C19—N3	111.8 (4)	C1—N5—C21	113.8 (4)
N7—C19—H19A	109.3	C2—N5—C21	113.6 (4)
N3—C19—H19A	109.3	C25—N6—C24	115.7 (6)
N7—C19—H19B	109.3	C20—N7—C19	111.5 (4)
N3—C19—H19B	109.3	C20—N7—C27	114.4 (4)
H19A—C19—H19B	107.9	C19—N7—C27	115.4 (4)
N7—C20—N4	113.5 (4)	C30—N8—C31	112.4 (6)
			
N2—C5—C6—C11	−162.1 (4)	C12—C5—N1—C3	12.7 (5)
N1—C5—C6—C11	−38.9 (6)	N2—C5—N1—C1	48.5 (6)
C12—C5—C6—C11	80.4 (6)	C6—C5—N1—C1	−76.3 (5)
N2—C5—C6—C7	26.4 (7)	C12—C5—N1—C1	157.8 (4)
N1—C5—C6—C7	149.6 (4)	N5—C1—N1—C3	87.6 (5)
C12—C5—C6—C7	−91.1 (5)	N5—C1—N1—C5	−52.9 (6)
C11—C6—C7—C8	−1.8 (8)	N4—C4—N2—C5	8.2 (5)
C5—C6—C7—C8	170.0 (5)	S1—C4—N2—C5	−175.2 (3)
C6—C7—C8—C9	1.8 (9)	N4—C4—N2—C2	163.9 (4)
C7—C8—C9—C10	−0.8 (10)	S1—C4—N2—C2	−19.5 (7)
C8—C9—C10—C11	−0.3 (9)	N1—C5—N2—C4	108.8 (4)
C7—C6—C11—C10	0.7 (8)	C6—C5—N2—C4	−125.8 (4)
C5—C6—C11—C10	−171.0 (5)	C12—C5—N2—C4	−1.1 (5)
C9—C10—C11—C6	0.4 (9)	N1—C5—N2—C2	−49.6 (5)
N2—C5—C12—N4	−5.9 (4)	C6—C5—N2—C2	75.7 (5)
N1—C5—C12—N4	−119.5 (4)	C12—C5—N2—C2	−159.5 (4)
C6—C5—C12—N4	116.3 (4)	N5—C2—N2—C4	−101.2 (5)
N2—C5—C12—N3	107.6 (4)	N5—C2—N2—C5	53.7 (6)
N1—C5—C12—N3	−6.0 (4)	N1—C3—N3—C12	10.3 (5)
C6—C5—C12—N3	−130.1 (4)	S2—C3—N3—C12	−173.6 (3)
N2—C5—C12—C13	−129.7 (4)	N1—C3—N3—C19	164.3 (4)
N1—C5—C12—C13	116.7 (4)	S2—C3—N3—C19	−19.6 (7)
C6—C5—C12—C13	−7.4 (5)	N4—C12—N3—C3	106.3 (5)
N4—C12—C13—C18	−31.2 (7)	C13—C12—N3—C3	−128.0 (4)
N3—C12—C13—C18	−155.3 (5)	C5—C12—N3—C3	−2.2 (5)
C5—C12—C13—C18	87.6 (6)	N4—C12—N3—C19	−51.3 (5)
N4—C12—C13—C14	150.7 (5)	C13—C12—N3—C19	74.4 (5)
N3—C12—C13—C14	26.6 (7)	C5—C12—N3—C19	−159.8 (4)
C5—C12—C13—C14	−90.4 (5)	N7—C19—N3—C3	−101.1 (5)
C18—C13—C14—C15	0.4 (9)	N7—C19—N3—C12	52.7 (5)
C12—C13—C14—C15	178.6 (5)	N2—C4—N4—C12	−12.5 (5)
C13—C14—C15—C16	−0.9 (10)	S1—C4—N4—C12	170.8 (3)
C14—C15—C16—C17	1.4 (11)	N2—C4—N4—C20	−155.4 (4)
C15—C16—C17—C18	−1.5 (11)	S1—C4—N4—C20	27.9 (6)
C14—C13—C18—C17	−0.5 (9)	N3—C12—N4—C4	−96.3 (4)
C12—C13—C18—C17	−178.6 (5)	C13—C12—N4—C4	138.1 (4)
C16—C17—C18—C13	1.1 (10)	C5—C12—N4—C4	11.3 (5)
N5—C21—C22—C26	−139.0 (6)	N3—C12—N4—C20	50.8 (5)
N5—C21—C22—C23	42.0 (7)	C13—C12—N4—C20	−74.8 (5)
C26—C22—C23—C24	0.0 (9)	C5—C12—N4—C20	158.5 (4)
C21—C22—C23—C24	179.1 (5)	N7—C20—N4—C4	89.0 (5)
C22—C23—C24—N6	0.5 (10)	N7—C20—N4—C12	−53.3 (5)
C23—C22—C26—C25	0.7 (9)	N1—C1—N5—C2	53.9 (6)
C21—C22—C26—C25	−178.4 (5)	N1—C1—N5—C21	−75.5 (6)
N6—C25—C26—C22	−2.0 (11)	N2—C2—N5—C1	−53.5 (6)
N7—C27—C28—C32	−43.3 (7)	N2—C2—N5—C21	76.0 (5)
N7—C27—C28—C29	140.3 (5)	C22—C21—N5—C1	−165.7 (4)
C32—C28—C29—C30	−0.2 (8)	C22—C21—N5—C2	66.4 (6)
C27—C28—C29—C30	176.3 (5)	C26—C25—N6—C24	2.3 (10)
C28—C29—C30—N8	−0.1 (9)	C23—C24—N6—C25	−1.6 (10)
N8—C31—C32—C28	0.0 (12)	N4—C20—N7—C19	52.5 (6)
C29—C28—C32—C31	0.3 (9)	N4—C20—N7—C27	−80.7 (5)
C27—C28—C32—C31	−176.3 (6)	N3—C19—N7—C20	−51.7 (5)
N3—C3—N1—C5	−14.4 (5)	N3—C19—N7—C27	81.0 (5)
S2—C3—N1—C5	169.5 (3)	C28—C27—N7—C20	−72.7 (5)
N3—C3—N1—C1	−156.2 (4)	C28—C27—N7—C19	155.9 (4)
S2—C3—N1—C1	27.7 (6)	C29—C30—N8—C31	0.4 (9)
N2—C5—N1—C3	−96.7 (5)	C32—C31—N8—C30	−0.3 (11)
C6—C5—N1—C3	138.6 (4)		

### Hydrogen-bond geometry (Å, °)

**Table:**

*D*—H···*A*	*D*—H	H···*A*	*D*···*A*	*D*—H···*A*
C33—H33···N8^i^	0.98	2.33	3.168 (9)	142 (8)

Symmetry codes: (i) −*x*+2, −*y*+1, −*z*+1.
